# Recycling Unused Midazolam Drug as Efficient Corrosion Inhibitor for Copper in Nitric Acid Solution

**DOI:** 10.3390/ma15082918

**Published:** 2022-04-16

**Authors:** Andrea Kellenberger, Delia Andrada Duca, Mircea Laurentiu Dan, Mihai Medeleanu

**Affiliations:** Faculty of Industrial Chemistry and Environmental Engineering, Politehnica University Timisoara, Piata Victoriei No. 2, 300006 Timisoara, Romania; andrea.kellenberger@upt.ro (A.K.); duca.delia@gmail.com (D.A.D.); mihai.medeleanu@upt.ro (M.M.)

**Keywords:** copper, corrosion, midazolam, expired drug, nitric acid

## Abstract

The current work explores the potential for recycling unused or expired Midazolam (MID) drug, a benzodiazepine derivative, as an efficient corrosion inhibitor for copper in nitric acid solution. The technical advantage of recycling expired MID drug relates to the avoidance of organic inhibitor production costs and the reduction of disposal costs of the expired medication. A combination of electrochemical methods (potentiodynamic polarization and electrochemical impedance spectroscopy), weight loss, and quantum chemical calculation were used to assess the inhibition mechanism and efficiency of MID. It was found that inhibition efficiency increases with inhibitor concentration, reaching a highest value of 92.9% for a concentration of 10^−4^ M MID. MID was classified as a mixed-type inhibitor, showing a preferential cathodic suppression mechanism. The obtained values of −45.89 kJ mol^−1^ for the standard free energy of adsorption indicate that the inhibition mechanism is based on chemisorption of MID molecules on the copper surface, which obeys the Langmuir isotherm. Surface analysis using scanning electronic microscopy revealed that MID offers high protection against corrosion during both immersion and polarization tests. Molecular modelling and quantum chemical calculations indicated chemical interactions between MID molecules and the copper surface, as well as electrostatic interactions. The results obtained using the different techniques were in good agreement and highlight the effectiveness of MID in the corrosion inhibition of copper.

## 1. Introduction

Corrosion of metals and alloys is a worldwide problem for natural and industrial environments. It has a negative effect on industrial equipment, on the durability of the infrastructure assets, and also on the quality of the environment. Thus, developing and applying corrosion protection methods to prevent financial losses in materials, equipment, and structures is necessary [1,2].

Copper and its alloys are industrially and domestically used, due to their good corrosion resistance, mechanical workability, malleability, and also high electrical and thermal conductivity [3,4,5,6,7]. In the standard electrode potential series, copper is a relative noble metal, in terms of its dissolution by hydrogen depolarization, and, therefore, it is sufficiently stable in acidic solutions with low oxidizing capacity. Nevertheless, copper is relatively sensitive if the solution contains oxidizing ions. However, copper corrosion may occur in the chemical cleaning processes of equipment with sulfuric acid, after the removal of the oxide layer [8,9,10].

Nitric acid is highly corrosive to copper and its alloys. Inhibiting the corrosive attack of metals in nitric acid is very difficult. One of the most popular and widely used methods of protecting metals and prolonging the life of equipment or installations, minimizing the consumption of natural resources, is the use of inhibitors [7,9,10]. Most acid inhibitors are organic compounds containing heteroatoms with lone pair electrons, such as P, S, N, O, functional groups, multiple bonds, and aromatic rings, which facilitates the adsorption of these molecules onto the metal surface [10,11,12,13,14]. The adsorption of the inhibitory structure occurs due to the difference in energy between the metal surface (lowest unoccupied molecular orbitals) and the non-participant electrons of the heteroatoms or π electrons (highest occupied molecular orbital) [15,16].

The copper corrosion process in nitric acid consists of Cu^2+^ ion production from the metal surface, which are transported into the bulk of the solution. In this aggressive media, the corrosion products (oxides and precipitates) are not able to form an adherent layer on the metal surface with a protective role, which leads to a continuous copper dissolution reaction. Many chemical compounds; organic and inorganic corrosion inhibitors, such as azole and its derivatives; Schiff bases; natural products; and noncompliant pharmaceutical drugs are used for copper protection in nitric acid media. The presence of heteroatoms in their structures, such as oxygen, sulfur, nitrogen, and phosphorus, facilitates the adsorption process onto the metal surfaces of the organic molecules or their oxidation product. Copper corrosion resistance in nitric acid decreases with an increase of the solution concentration, and the relation between the inhibitor concentration, organic molecule adsorption, and corrosion rate are the overall key processes [9].

Benzodiazepines have been considered in many areas, such as medicinal chemistry and pharmacology, due to their anti-inflammatory, analgesic, anticonvulsant, and sedative properties [17,18,19,20]. Over the past decade, the inhibitive action of benzodiazepine has attracted the attention of many researchers [17,21,22,23,24]. In economic and environmental terms, they are non-toxic and environmental-friendly compounds when used as corrosion inhibitors, because of their alternative use minimizes the environmental pollution when they are improperly stored or disposed of by incineration, and also reduces their elimination costs [25,26]. MID drug, a benzodiazepine derivative, has aromatic rings in its structure, as well as multiple bonds, and atoms of nitrogen, fluorine and chlorine, which contain lone pairs of electrons, being an ideal candidate for a corrosion inhibitor. It is not economically viable to use the fresh drug because it is more expensive than the organic inhibitors currently used, which is why this research has focused on the use of expired midazolam [15,26]. MID has been used in large quantities during the pandemic of recent years, being listed by the Royal College of Anesthetists as a “first-line” sedative in the management of COVID-19 patients. Consequently, considerable quantities of this drug were manufactured in 2020, and after medical studies identified more effective sedative drugs, whole batches of MID remained unused and will soon expire [27,28,29].

In this work, the inhibition efficiency of MID for copper corrosion in nitric acid is investigated. Expired MID drug is used as a non-toxic alternative to traditional inhibitors, also demonstrating the possibility of recycling such expired drugs. A quantitative assessment of the inhibition efficiency was made using various methods, including weight loss and polarization measurements, as well as electrochemical impedance spectroscopy. The surface morphology of corroded samples in the absence and presence of various concentrations of MID was investigated by field emission scanning electron microscopy, demonstrating its effectiveness for corrosion protection of copper.

## 2. Materials and Methods

### 2.1. Materials 

**Figure 1:**
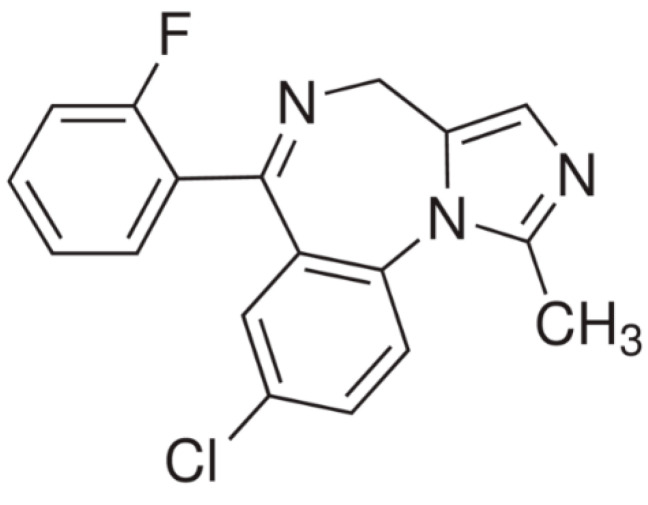
Chemical structure of Midazolam.

MID is a benzodiazepine derivative, which belongs to the pharmaceutical group of hypnotics and sedatives, and it is used as a short term acting hypnotic. MID (C_18_H_13_ClFN_3_) or 8-chloro-6-(2-fluorophenyl)-1-methyl-4*H*-imidazo[1,5-a][1,4]benzodiazepine is a crystalline compound, with the chemical structure given in Figure 1.

When formulated as a solution for injection, Midazolam 5 mg/mL contains 5.56 mg midazolam hydrochloride (equivalent to 5 mg midazolam) and the excipients sodium chloride, hydrochloric acid 1N, and water. The solubility of MID is pH dependent, with values below 0.1 mg mL^−1^ at neutral pH, which increase significantly at acid pH. This is explained by the reversible hydrolysis of MID at pH < 4, when the benzodiazepine ring is opened to form the corresponding benzophenone [30]. Thus, a primary amino group is formed, which gives MID the ability to form highly-water soluble salts with acids [31].

The corrosion test solution was prepared by diluting analytical grade HNO_3_ 65% (AnalytiCals Carlo Erba, Val de Reuil, Normandie, France) in distilled water. Five different concentrations of MID, varying from 10^−4^ to 10^−6^ M, were prepared in 0.1 M HNO_3_ solution using corresponding amounts of expired Midazolam solution for injection.

Weight loss measurements were carried out on disc-shaped metallic copper (99%) samples, with the dimensions Φ 15 mm × 5 mm. Prior to all tests, the copper samples were cleaned by mechanical grinding with SiC paper (grain size 800 to 2400), followed by polishing with diamond suspension (DiaDuo Struers, Cleveland, OH, USA), with a grain size of 6 and 3 µm, respectively. Finally, the samples were thoroughly washed with distilled water, ultrasonically cleaned in acetone, and dried.

### 2.2. Weight Loss Measurements

For the weight loss measurements, the cleaned and weighed copper samples were immersed in 150 mL of the test solutions: 0.1 M HNO_3_ solution without inhibitor (blank) and with different concentrations of the inhibitor, ranging from 10^−4^ to 10^−6^ M, at room temperature. After an exposure time of 48 h, the specimens were taken out, cleaned to remove the corrosion products, and weighed again. The weight loss was expressed in g m^−2^ h^−1^ by dividing by the total surface area of specimens and the exposure time. The inhibition efficiency (*E*) and surface coverage (*θ*) of MID were obtained using Equations (1) and (2):

*E* (%) = (1 − *W*_corr_/*W*^o^_corr_) × 100 (1)

*θ* = 1 − *W*_corr_/*W*^o^_corr_ (2)

where *W*_corr_ and *W*^o^_corr_ represent the weight loss of the samples in the presence and absence of the inhibitor, respectively.

### 2.3. Electrochemical Methods

All electrochemical measurements were performed in a standard three-electrode configuration electrochemical cell connected to an Autolab PGSTAT 302N potentiostat/galvanostat (Metrohm Autolab, Utrecht, The Netherlands). The working electrode consisted of an 8-mm diameter copper rod, isolated in a heat shrink tubing, leaving exposed a geometric surface area of 0.5 cm^2^. Two graphite rods were used as counter electrodes and the reference was a silver/silver chloride Ag/AgCl (3M KCl, Metrohm Autolab, Utrecht, The Netherlands) electrode. Before measurements, the samples were stabilized in the test solutions for 30 min, and the open circuit potential (OCP) values were measured. Afterward, electrochemical impedance spectroscopy (EIS) measurements were carried out at the OCP value, in the frequency range from 10^−2^ to 10^5^ Hz and AC voltage amplitude of 10 mV rms. A logarithmic distribution of 10 points per decade was used to collect 60 points for each spectrum. Using the ZView 3.0 software (Scribner Associates, Inc., Southern Pines, NC, USA), the experimental EIS data were fitted to the equivalent electrical circuit (EEC) using a complex non-linear least squares Levenberg–Marquardt procedure. Finally, potentiodynamic polarization curves were obtained at a scan rate of 1 mV s^−1^ in the potential range of −250 mV to +250 mV relative to the OCP value. Reproducibility was checked by repeating the polarization curves three times. Corrosion parameters, such as corrosion potential *E*_corr_, corrosion current *i*_corr_, and corrosion rate *v*_corr_ were determined for the copper samples in the blank solution and in the presence of different concentrations of MID.

### 2.4. Surface Morphology

After both weight loss and polarization measurements, the structure and morphology of the copper samples were examined using field emission scanning electron microscopy (FE-SEM) with a QUANTA FEG 250 microscope (FEI, Hillsboro, OR, USA). FE-SEM images were taken using the secondary electrons detector, at a accelerating voltage of 30 kV and with a working distance of 10 mm.

### 2.5. Molecular Modelling and Quantum Chemical Calculation

Quantum chemical calculations were performed using GAUSSIAN 09 software (GAUSSIAN 09, Revision B.01, Gaussian, Inc., Wallingford CT, USA, 2010) [32]. The geometry of MID molecule was fully optimized in a vacuum and water environment, applying the density functional theory method with restricted B3LYP hybrid functional, with the 6-31G(d) basis set. Some molecular descriptors, such as energies of highest occupied molecular orbital (HOMO), lowest unoccupied molecular orbital (LUMO), HOMO-LUMO gap, dipole moment, hardness, and softness, were calculated to describe the interaction between midazolam and copper.

## 3. Results and Discussion

### 3.1. Weight Loss Measurements

Weight loss measurements were performed in 0.1 M HNO_3_ solution, in the absence and presence of different concentrations of MID, for an exposure time of 48 h. The calculated values of corrosion rate expressed in (g m^−2^ h^−1^), inhibition efficiency and surface coverage, obtained with Equations (1) and (2), are summarized in Table 1. The obtained results indicate that the inhibition efficiency depends on MID concentration, and reached the maximum value at a concentration of 10^−4^ M.

The inhibition mechanism of MID can be explained by adsorption of inhibitor molecules on the copper surface, which blocks the active corrosion sites and reduces the corrosion rate. At higher MID concentrations, the number of inhibitor molecules adsorbed at the copper–solution interface increases; thus, increasing the inhibition efficiency. The adsorption of MID on the copper surface is ensured by the interaction with the lone pair of electrons of the nitrogen atom. The vacant d orbitals of the copper atom are prone to forming coordinative bonds with electron donor atoms.

### 3.2. Potentiodynamic Polarization Measurements

**Figure 2:**
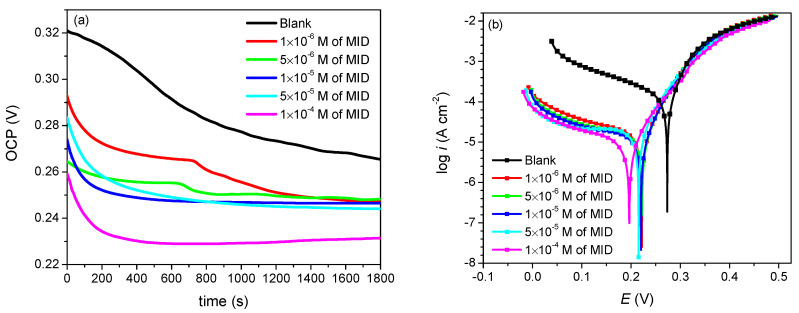
(**a**) Open circuit potential over time and (**b**) potentiodynamic polarization curves of copper in 0.1 M HNO_3_, in the absence and presence of different concentrations of MID.

Figure 2 shows the open circuit potential evolution during 30 min of immersion and the potentiodynamic polarization curves obtained on copper in 0.1 M HNO_3_ solution, in the absence and presence of various concentrations of MID. The corrosion parameters (*E*_corr_, *i*_corr_, and *v*_corr_) were obtained by the Tafel extrapolation method, and the polarization resistances (*R_p_*) were calculated from the Stern–Geary equation, according to Equation (3). All corrosion parameters are given in Table 2.

*R*_p_ = *b*_a_·*b*_c_/[*i*_corr_·2.303·(*b*_a_ + *b*_c_)] (3)

where *R*_p_ is the polarization resistance, Ω cm^2^; *b*_a_ and *b*_c_ represent the anodic and cathodic Tafel slopes; and V and *i*_corr_ is the corrosion current, A cm^−2^.

The open circuit potential values represented in Figure 2a show a shift to more negative values with increasing immersion time, both in the presence and absence of the inhibitor. The OCP values in the presence of MID were more negative than in the blank solution, suggesting that the inhibitor molecules have the ability to adsorb and block the active sites on the copper surface. Under these circumstances, a cathodic reaction takes place with high overpotential; thus, shifting the corrosion potential to more negative values. The potentiodynamic polarization curves from Figure 2b clearly point to a significant decrease of the cathodic currents and a less important decrease of the anodic currents with increasing concentrations of MID, indicating that MID molecules preferentially inhibit the cathodic processes. The quantitative data from Table 2 show that the effect of increasing concentrations of MID is to drastically decrease the corrosion current and corrosion rate, as compared to the values recorded in the blank solution. The inhibition efficiency follows the same trend, increasing with the increase of the concentration of inhibitor, reaching a highest value of 92.9% for a concentration of 10^−4^ M of MID. In addition, the corrosion potential *E*_corr_ shifts to more negative values in the presence of MID, with a maximum change of 75 mV at the highest concentration of MID. The difference between *E*_corr_ in the presence and absence of the inhibitor is often used to classify inhibitors as anodic, cathodic, or mixed-type, considering that an offset lower than 85 mV indicates mixed-type inhibitors, while an offset higher than 85 mV describes anodic and cathodic inhibitors [3,33,34,35,36]. Since the maximum shift in the present study was 75 mV, MID can be classified as a mixed-type inhibitor, meaning it can suppress both cathodic and anodic processes involved in copper corrosion.

Analysis of the anodic and cathodic Tafel slopes gives indications about the corrosion inhibition mechanism. In the presence of MID, the cathodic branch of the polarization curve shows a region of activation-controlled hydrogen evolution reaction, followed by a diffusion-controlled reduction reaction of dissolved oxygen. The cathodic current associated with oxygen reduction decreases gradually as the concentration of MID increases. Moreover, the cathodic Tafel slope increases, involving a strong interference of MID molecules in the oxygen reduction mechanism. In this situation, the corrosion rate is controlled by the rate of cathodic reaction. On the anodic branch of the polarization curves, two distinct regions are observed: near the corrosion potential a region with a rapid increase of the current, characterized by a typical Tafel behavior, due to the anodic dissolution of copper; followed by a region with a diffusion limited current. The anodic Tafel slope increases with MID concentration, which demonstrates that copper dissolution is also hindered in the presence of MID. According to the Pourbaix diagram for the copper-water system [37], copper is corroded to Cu^2+^ in strong acid media, while the formation of protectives oxides of copper is only possible in weakly acidic and alkaline electrolytes. As a result, the corrosion of copper in HNO_3_ occurs directly, in a single stage, without the formation of a passivating oxide layer [10].

All these observations indicate the effectiveness of MID in the corrosion inhibition of copper. Based on the Tafel polarization results, it was concluded that MID can be considered a mixed-type adsorption inhibitor, acting preferentially on the cathodic oxygen reduction reaction.

### 3.3. Electrochemical Impedance Spectroscopy Measurements

**Figure 3:**
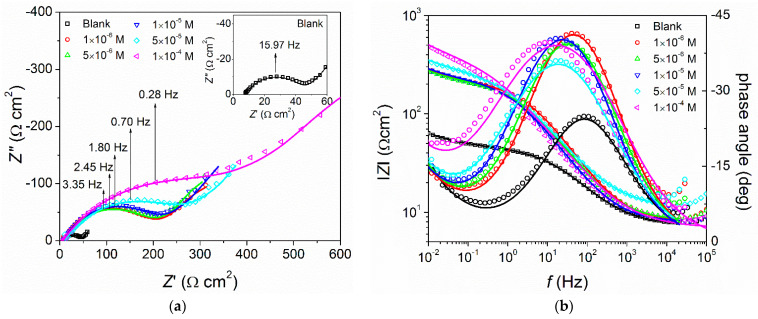
(**a**) Nyquist and (**b**) Bode plots of copper in 0.1 M HNO_3_, in the absence and presence of different concentrations of MID. Open symbols show experimental values and continuous lines were obtained by fitting.

The inhibition effect of MID on Cu corrosion in 0.1 M HNO_3_ solution was investigated using electrochemical impedance spectroscopy measurements. Figure 3 shows Nyquist and Bode plots of the impedance determined in the presence of different concentrations of MID, as well as without the inhibitor, in 0.1 M HNO_3_ solution, 30 min after the immersion, at the open circuit potential value. In Figure 3, the continuous lines were generated by fitting, using a Randles circuit, while open symbols indicate experimental data.

The shape of the Nyquist plots in Figure 3a is characterized by the presence of a high frequency semicircle, corresponding to the charge-transfer resistance (*R*_ct_) in parallel with the double-layer capacitance (*C*_dl_), followed by a straight line at low frequencies, with a slope of approximately-1, related to a Warburg impedance, which describes a diffusion process. Literature data report that the diffusion process indicated by the Warburg impedance can be related to the diffusion of Cu^2+^ ions from the electrode surface to the bulk solution or to the diffusion of dissolved oxygen from the bulk to the interface [38]. Since the Warburg impedance does not disappear in the presence of MID, this indicates that, both in the presence and absence of MID, the corrosion of copper is under mixed control of both charge-transfer and diffusion processes. The diameter of the high frequency loop represents the *R*_ct_ value, which obviously increases with MID concentration, indicating that charge-transfer is inhibited in the presence of MID. This is also evident from the shift of the characteristic frequency of the charge-transfer process to lower values with increasing MID concentration. The Bode plots presented in Figure 3b clearly evidence that the magnitude of impedance at low frequency increases almost one order of magnitude for the highest concentration of MID, as compared to the blank solution. Similarly, the phase angle in the presence of MID increases, and the frequency of the maximum shifts to lower values, confirming the inhibitor ability of MID.

The EEC used to assess the impedance parameters is a Randles circuit, consisting of a serial connection between the solution resistance *R*s and a parallel connection of the double-layer capacitance *C*_dl_, and the charge-transfer resistance *R*_ct_ in series with a Warburg impedance. Frequently, real systems deviate from the ideal capacitive behavior; thus, the capacitance is typically substituted by a constant phase element (CPE), whose impedance is given by Equation (4):

*Z*_CPE_ = 1/*T* (j*ω*)*^n^* (4)

where *T* is a parameter related to the double-layer capacitance and *n* is an exponent between 0 and 1 that describes the CPE’ constant phase angle. The true double-layer capacitance values may be estimated according to Equation (5) [39]:

*T* = *C*_dl_*^n^* (*R*_S_^−1^ + *R*_ct_^−1^)^1^^−*n*^ (5)

The impedance of the finite length Warburg element, corresponding to situations when the diffusion layer is bounded and not infinite, is defined by Equation (6):

*Z*_W_ = *R*_d_ tanh (j*ωτ*_d_)^1/2^*/*(j*ωτ*_d_)^1/2^ (6)

where *R*_d_ is the diffusion resistance (Ω cm^2^) and *τ*_d_ is the diffusion time constant (s), given as the ratio between the squared diffusion layer thickness and the diffusion coefficient.

This model was chosen to reflect the processes taking place at the electrode surface, the charge-transfer reaction described by the *R*_ct_-CPE connection and the diffusion of Cu^2+^ and/or dissolved oxygen given by the Warburg element. The impedance parameters obtained by modelling the experimental data are listed in Table 3, together with their errors and the goodness of fit; expressed by the Chi-squared values. The low values of obtained errors and Chi-squared describe a good quality of fit of the experimental data with the proposed model. The inhibition efficiency was than calculated with Equation (7), using the determined charge-transfer values in the absence (*R*^o^_ct_) and presence (*R*_ct_) of the inhibitor.

*E* (%) = (1 − *R*^o^_ct_/*R*_ct_) × 100 (7)

The obtained results show that the charge-transfer resistance has an increasing tendency with the increasing of inhibitor concentration, which corresponds to a reduction of the corrosion rate. Furthermore, the diffusion resistance and the diffusion time constant have higher values in the presence of MID, as compared to the blank, meaning that MID molecules also interfere with the diffusion of ions from the solution to the copper surface, by forming a barrier that slows down this process. The evidence that MID molecules adsorb on the copper surface is given by the double-layer capacitance values, which gradually decrease in the presence of the inhibitor. By adsorption at the copper–solution interface, the MID molecules replace the adsorbed water molecules and create a protective inhibitor film, which explains the decrease of *C*_dl_ values. Generally, the capacitance of a parallel plate capacitor separated by a dielectric medium is given by Equation (8):

*C* = *ε*_o_·*ε*_r_·*S*/*d* (8)

where *ε*_o_ is the vacuum permittivity, *ε*_r_ is the relative permittivity, *S* the surface area, and *d* represent the dielectric thickness.

If the double-layer at the copper/solution interface is treated as a capacitor, where the dielectric medium is represented by the adsorbed molecules, a decrease of the double-layer capacitance can be explained in terms of a decrease in the local dielectric constant, and/or an increase in the double-layer thickness. This is the case when bulky inhibitor molecules, with lower dielectric constant than water, adsorb and replace the water molecules from the interface.

The inhibition efficiency calculated from impedance data is close to the values determined from the weight loss and polarization measurements.

### 3.4. Adsorption Isotherm

Valuable information about the nature of interactions between the organic inhibitor molecule and the metal surface can be obtained from the adsorption isotherm. The experimental data obtained for the corrosion of copper in nitric acid are best described by the Langmuir adsorption isotherm, given by Equation (9):

*c*_inh_/*θ* = 1/*K*_ads_ + *c*_inh_ (9)

where *c*_inh_ represents the concentration, *θ* is the degree of the surface coverage, and *K*_ads_ stands for the equilibrium constant of the adsorption process.

**Figure 4:**
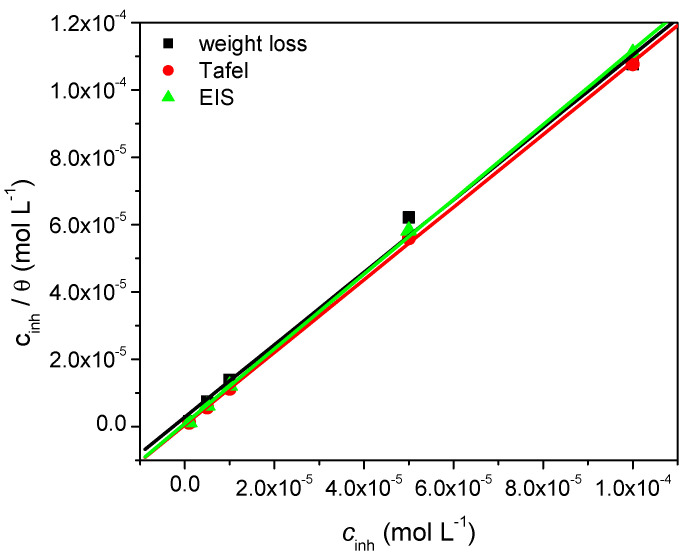
Langmuir adsorption isotherms of MID on copper in 0.1 M HNO_3_ solution obtained from weight loss, Tafel, and EIS measurements.

Figure 4 shows the linear dependence of *c*_inh_/*θ* versus *c*_inh_, obtained by using experimental data computed from weight loss, Tafel, and EIS measurements, respectively. In all cases a straight line is obtained, with correlation coefficient (*R*^2^) values above 0.99 and a slope closed to the unit. This is an evidence that the adsorption process of MID on copper surface obeys the Langmuir isotherm.

The adsorption constant *K*_ads_ determined from the intercept of the straight lines allows calculating the standard free energy of adsorption (Δ*G*^o^_ads_) using Equation (10):

Δ*G*^o^_ads_ = −R*T* ln(55.5·*K*_ads_) (10)

where R is the universal gas constant (8.314 J mol^−1^ K^−1^), *T* is the thermodynamic temperature in K, and 55.5 represents the molar concentration of water in the solution. Table 4 summarizes the thermodynamic parameters related to MID adsorption on the copper surface.

The calculated values of Δ*G*^o^_ads_ point to the existence of a chemical interaction between the copper surface and the inhibitor molecules. Similar values of Δ*G*^o^_ads_, indicating strong adsorption by a chemical mechanism, have also been found for other benzodiazepine derivatives acting as corrosion inhibitors for copper [3] or mild steel [20,40], as well as for benzimidazole derivatives [41].

### 3.5. Surface Analysis

**Figure 5:**
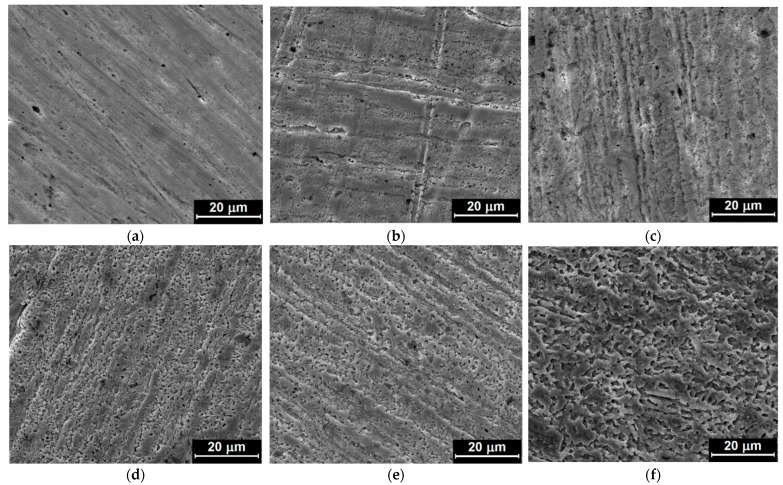
FE–SEM images of copper surface after weight loss measurements conducted for 48 h in 0.1 M HNO_3_ solution with different concentrations of the inhibitor: (**a**) 10^−4^ M MID; (**b**) 5 × 10^−5^ MID; (**c**) 10^−5^ MID; (**d**) 5 × 10^−6^ MID; (**e**) 10^−6^ MID; and (**f**) without inhibitor.

The surface morphology of copper samples was investigated using FE-SEM after 48 h of corrosive attack during the weight loss measurements. Figure 5a–e shows FE–SEM images of the copper surface in the presence of different concentrations of the inhibitor; and in Figure 5f, in the absence of the inhibitor.

A strong corrosive attack is observed in nitric acid solution without inhibitor, with clear pits and cavities, whose dimensions decrease as the inhibitor concentration increases, while at the maximum concentration of the inhibitor the copper sample was protected, due to the adsorption of MID on the copper surface. The inhibitor forms a protective film that can be seen as a physical barrier, which slows down the transport of particles involved in the corrosion process; thus, reducing the corrosion rate. It is established that for the maximum concentration of the inhibitor (Figure 5a), there are practically no signs of corrosion. With the decrease of the inhibitor concentration (Figure 5b,c), it is noticed that the corrosive attack occurs mainly on the edges formed during the metallographic preparation process of the sample. For the lowest MID concentrations (Figure 5d,e), the corrosive attack begins to advance in depth, and the proportion of dissolved and corroded areas increases. This is evidence that MID concentration plays an important role in the inhibition process.

**Figure 6:**
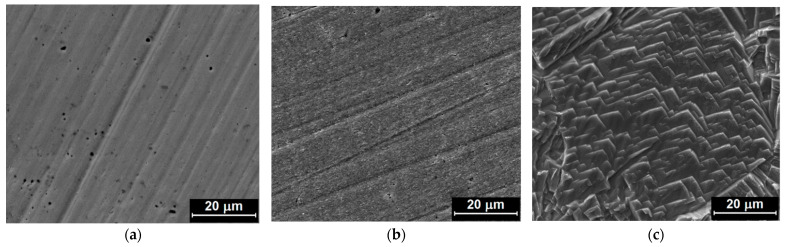
FE–SEM images of the copper surface: (**a**) before and after potentiodynamic polarization measurements in 0.1 M HNO_3_ solution with (**b**) 10^−4^ M MID and (**c**) without inhibitor.

Furthermore, the surface morphology of copper samples after corrosion during potentiodynamic polarization measurements was analyzed by FE-SEM. Figure 6 presents FE–SEM images of the metallic copper sample before and after the potentiodynamic polarization measurements, in the presence of the highest concentration of MID, as well as in the absence of the inhibitor.

Corrosion during anodic polarization induces a different type of damage as that observed during immersion, since the potential is swept to more positive values with the corrosion potential, so copper dissolution takes place at a higher rate. Without inhibitor, the Cu surface was heavily etched, preferentially along edges and corners, leading to a rectangular shaped, stepped morphology, depending on the crystallographic orientation of the corroded grains. A similar preferential etching has been observed for Cu corrosion in 100 mM HNO_3_ in solution purged with a high oxygen content [42]. In the presence of MID, the Cu surface looks slightly rougher than the metallic Cu surface, with the original polishing lines clearly visible, indicating that the MID efficiently inhibited corrosion and reduced the copper dissolution rate.

### 3.6. Quantum Chemical Calculation

**Figure 7:**
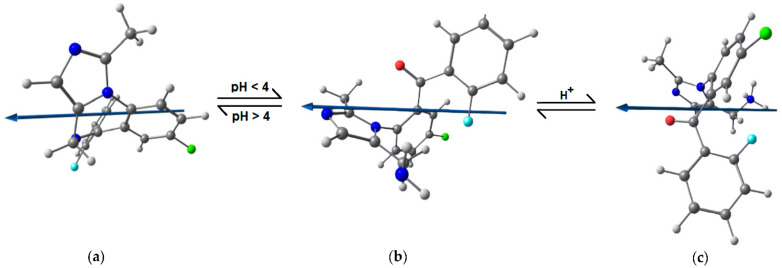
Optimized molecular structures of MID molecule in water: (**a**) closed-ring structure; (**b**) open-ring structure resulting by reversible hydrolysis in acid media; and (**c**) open-ring structure protonated at the free amino group. The arrow shows the orientation of the dipole moment. Atoms are depicted in: blue—nitrogen, green—chlorine, cyan—fluorine, red—oxygen, dark grey—carbon, light grey—hydrogen.

To provide a further understanding of the experimentally observed behavior of MID at the copper–solution interface, molecular modelling was performed. Literature data report that in acid media at pH < 4, MID undergoes a reversible hydrolysis [30], when the benzodiazepine ring is opened, resulting in the corresponding ketone form and a free primary amino group, which can be further protonated. To reflect these changes and the true state in which MID molecules exist in 0.1 M HNO_3_, Figure 7 shows the molecular models of the three forms of MID molecule in water: the closed-ring structure, the open-ring structure, and its protonated form.

Quantum chemical parameters used to explain the inhibition efficiency of MID, such as the HOMO and LUMO energy levels, the HOMO–LUMO energy gap (ΔE), and the dipole moment (µ) are summarized in Table 5 for the three structures of MID molecule. Additionally, ionization potential (*I* = −*E*_HOMO_), electron affinity (*A* = −*E*_LUMO_), absolute electronegativity (*χ*) absolute hardness (*η*), and softness (*σ*) defined according to Pearson [43], as well as the fraction of transferred electrons (Δ*N*) were calculated using Equations (11)–(14) and are also listed in Table 5.

*χ* = (*I* + *A*)/2 (11)

*η* = (*I* − *A*)/2 (12)

*σ* = 1/*η* (13)

Δ*N* = (*χ*_Cu_ − *χ*_inh_)/[2·(*η*_Cu_ + *η*_inh_)] (14)

Considering the frontier orbital molecular theory, the reactivity is a direct consequence of HOMO–LUMO interactions. The energy of HOMO relates to the ionization potential and describes the electron donor properties of a molecule, while the energy of LUMO relates to electron affinity and characterizes the electron accepting ability of a molecule. The computed values of the HOMO energy for MID closed-ring and open-ring structures are −6.039 and −5.799 eV, which gives an ionization potential of *I*_MID_ of 6.039 and 5.799 eV, respectively. When compared to the ionization potential of copper, *I*_Cu_ = 7.726 eV, this predicts that electrons are more easily transferred from the MID molecule to the vacant d-orbitals of copper. Moreover, the up-shift of the *E*_HOMO_ for the open-ring MID structure signifies a better ability of the molecule to donate electrons.

The HOMO–LUMO energy gap is regarded as a descriptor of a molecule’s chemical stability, with large Δ*E* values indicating greater chemical hardness and, hence, a low reactivity, in contrast to low Δ*E* values, meaning lower chemical hardness; thus, a high reactivity. Consequently, high lying HOMO and low lying LUMO, and, hence, low Δ*E* values, are frequently correlated with superior inhibition efficiency [44]. Comparing the quantum chemical parameters of the open-ring and closed-ring structures of MID, it is clear that the HOMO energy shifts up and the LUMO energy shifts down for the ketone form, resulting in lower Δ*E* values than the closed-ring structure, implying a higher reactivity and, thus, stronger interactions with the copper surface. For the protonated form, the down-shift of HOMO is compensated by the down shift of LUMO, so that the energy gap Δ*E* and absolute hardness are nearly identical to that of the closed-ring MID structure.

The absolute electronegativity *χ* is also useful in predicting chemical reactivity. When two molecules react, the electrons will be partially transferred from the molecule with the highest chemical potential (low *χ*) to that of lowest chemical potential (high *χ*) [43], until the chemical potentials become equal. The absolute electronegativity of MID was calculated to 3.866 eV (closed-ring) and 4.007 eV (open-ring), respectively, which are lower than the absolute electronegativity of Cu (4.48 eV). This indicates that electrons will be transferred from MID to copper, in accordance with the calculated values of the free energy of adsorption Δ*G*^o^_ads_, which point to the formation of chemical bonds between the inhibitor and copper. The low difference in electronegativity between copper and the protonated MID form translates into a Δ*N* value close to zero, showing that protonation of free NH_2_ group deactivates it for chemisorption.

Furthermore, the absolute hardness and softness of a molecule are indicators of the chemical reactivity. Soft molecules, with a low HOMO–LUMO energy gap, are more reactive than hard molecules, having a low resistance to electron transfer. Hard molecules, with a high HOMO–LUMO energy gap, oppose a higher resistance to changes in their electron number and distribution. For MID, the open-ring structure has lower values of absolute hardness, *η*_MID_ = 1.793 eV and higher softness *σ*_MID_ = 0.558 eV^−1^ than the closed-ring structure. This indicates that it is a soft molecule, with increased tendency to transfer electrons to copper, as also shown by the fraction of transferred electrons Δ*N* = 0.132. The protonated form has a similar hardness and softness to the closed-ring structure, but due to higher electronegativity the Δ*N* value is nearly zero, because the electron pair of the N atom is already involved in a coordinate bond with protons. However, it must be taken into account that, during corrosion, the oxygen reduction reaction leads to a local pH increase at the interface, which might cause deprotonation, and MID could exist in the open-ring neutral form.

Electrostatic interactions are also possible between MID and the copper surface, in addition to chemical interactions. This can be quantified by the dipole moment, which is an indicator of the charge separation or electronic distribution in a molecule. Corrosion studies generally report that molecules with higher dipole moments are expected to show enhanced adsorption on the metal surface, and to have a higher inhibition efficiency [44,45]. The calculated dipole moment of MID (4.653 D) shows a significant increase after hydrolysis (8.600 D) and protonation (16.414 D). All these values are higher than that of water (1.85 D), which is also an indication about the ability of MID to strongly interact with the copper surface. All the evaluated quantum chemical parameters point to the ability of MID to adsorb on the copper surface by replacing previously adsorbed water molecules.

## 4. Corrosion Inhibition Mechanism

MID molecules adsorbs on the copper surface by electron transfer from MID to the empty, low-energy d orbitals of copper. The formation of such donor–acceptor complexes between free electrons of the inhibitor and a vacant d orbitals of the metal is responsible for the suppression of the corrosion process [34]. Since the polarization curves showed that MID acts as a mixed type inhibitor, predominantly blocking the cathodic process, it is expected that MID will preferentially adsorb on the cathodic sites. In acid solutions, the benzodiazepine ring is opened by a reversible hydrolysis, resulting in a carbonyl and a primary amino group. Hence, N atoms from the amino group and the imidazole ring, having lone pairs of electrons and/or the π electrons of the imidazole ring, could be responsible for the adsorption on the copper surface. In acidic solution, MID can be protonated at the free amino group, according to Equation (15). Protonation changes the electron density of MID and makes the amino group unavailable for chemisorption, so MID molecules will be oriented with the electron rich regions toward the copper surface. Both neutral and protonated forms of MID are highly polar molecules, so when they are present at the metal–solution interface they will compete with water or nitrate molecules for the available adsorption sites. MID adsorption occurs via Equation (16), by replacing adsorbed water molecules.

MID + *x*H_3_O^+^ ⇌ [MID − H*_x_*]*^x^*^+^ + *x*H_2_O (15)

[MID − H*_x_*]*^x^*^+^ + *n*H_2_O_(ads)_ ⇌ [MID − H*_x_*]*^x^*^+^ _(ads) +_
*n*H_2_O (16)

All the experimental measurements and theoretical calculations confirm that MID is an effective inhibitor for copper corrosion in HNO_3_ solution.

To have a clearer overview of the inhibition effect of MID on copper corrosion, Table 6 shows a comparison of different parameters related to the inhibition efficiency for selected organic molecules, including some drugs, for copper corrosion in different corrosive media.

The results obtained in this work regarding the inhibition efficiency of MID on Cu corrosion are within the range reported in literature. A comparison should also take into account the inhibitor concentration and corrosive test solution. Nevertheless, in terms of corrosion current, polarization resistance, and inhibition efficiency, MID containing a fused imidazole-benzodiazepine ring is superior to levetiracetam drug containing a pyrrole ring, and similar to metronidazole drug, which is an imidazole derivative. This study demonstrates the efficiency of MID in the corrosion inhibition of copper and opens up the possibility of recycling such unused or expired drugs, as a non-toxic alternative to traditional inhibitors.

## 5. Conclusions

Midazolam drug was found to be an effective corrosion inhibitor for copper in nitric acid solution, reaching an inhibition efficiency of 92.9% for a concentration of 10^−4^ M. The results of the potentiodynamic polarization measurements indicated that MID is a mixed-type inhibitor, exhibiting a preferential cathodic suppression mechanism. The inhibition mechanism determined from thermodynamic calculation was chemisorption, following the Langmuir adsorption isotherm. EIS measurements showed that the presence of MID in the corrosive media improved the corrosion resistance of copper, as indicated by increased charge-transfer and diffusion resistances. Another effect evidenced by EIS measurements, is the decrease of the double-layer capacitance, due to adsorption of MID molecules on the copper surface and substitution of previously adsorbed water molecules. The results of FE-SEM imaging reflect that the inhibiting properties depend on the inhibitor’s concentration, indicating a high degree of protection at concentrations of 10^−4^ M. All the evaluated quantum chemical parameters clearly indicate a high tendency of MID to transfer electrons to the vacant d-orbitals of copper, forming chemical bonds, which explains the development of a highly protective inhibitor film.

## Figures and Tables

**Table 1:** Corrosion rate expressed in (g m^−2^ h^−1^), inhibition efficiency and surface coverage values obtained from weight loss measurements for copper in 0.1 M HNO_3_ in the absence and presence of different concentrations of MID, after an exposure time of 48 h.

Concentration (M)	*W*_corr_ (g m^−2^ h^−1^)	*E* (%)	*θ*
Blank	0.255	-	-
1 × 10^−6^	0.123	51.8	0.518
5 × 10^−6^	0.084	67.1	0.671
1 × 10^−5^	0.075	70.6	0.706
5 × 10^−5^	0.050	80.4	0.804
1 × 10^−4^	0.018	92.9	0.929

**Table 2:** Corrosion parameters obtained by Tafel extrapolation method and inhibition efficiencies for copper in 0.1 M HNO_3_, in the absence and presence of different concentrations of MID.

Concentration (M)	*E*_corr_ (V)	*i*_corr_ (µA cm^−2^)	*b*_a_ (mV)	*b*_c_ (mV)	*R*_p_ (Ω cm^2^)	*v*_corr_ (mm y^−1^)	*E* (%)	*θ*
Blank	0.273	123.1	39	−220	117	1.43	-	-
1 × 10^−6^	0.222	15.4	54	−328	1306	0.18	87.5	0.875
5 × 10^−6^	0.223	14.2	57	−390	1426	0.16	88.5	0.885
1 × 10^−5^	0.220	13.1	55	−410	1602	0.15	89.3	0.893
5 × 10^−5^	0.215	13.0	55	−217	1456	0.15	89.4	0.894
1 × 10^−4^	0.196	8.7	63	−262	2540	0.10	92.9	0.929

**Table 3:** Impedance parameters for copper in 0.1 M HNO_3_ in the absence and presence of varying concentrations of MID calculated by fitting the experimental data.

Concentration (M)	*R*_S_ (Ω)	CPE-T (F cm^−2^ s^n−1^)	*n*	*R*_ct_ (Ω cm^2^)	*C*_dl_ (µF cm^−2^)	*R*_d_ (Ω cm^2^)	*τ*_d_ (s)	*Chi^2^*	*E* (%)
Blank	7.4 (0.5%)	1.53 × 10^−3^ (2.3%)	0.59	38.6 (0.6%)	82.4	61.5 (2.0%)	107.7	6.1 × 10^−4^	-
1 × 10^−6^	8.1 (0.7%)	6.34 × 10^−4^ (1.8%)	0.66	191.5 (1.1%)	43.4	291.2 (2.7%)	167.3	6.9 × 10^−4^	79.9
5 × 10^−6^	8.2 (0.8%)	8.64 × 10^−4^ (2.0%)	0.63	193.9 (0.9%)	47.7	296.2 (2.7%)	170.2	1.2 × 10^−3^	80.1
1 × 10^−5^	7.9 (0.8%)	1.08 × 10^−3^ (2.1%)	0.60	217.4 (1.5%)	45.8	394.6 (7.5%)	272.0	1.3 × 10^−3^	82.3
5 × 10^−5^	8.1 (0.7%)	1.27 × 10^−3^ (1.7%)	0.56	274.0 (1.5%)	45.6	501.6 (2.4%)	280.0	7.9 × 10^−4^	85.9
1 × 10^−4^	6.9 (0.9%)	1.44 × 10^−3^ (1.6%)	0.55	388.5 (1.9%)	35.6	828.4 (4.2%)	307.7	1.2 × 10^−3^	90.1

**Table 4:** Thermodynamic parameters for MID adsorption on copper in 0.1 M HNO_3_ solution.

Measurement	*R* ^2^	*K*_ads_ (M^−1^)	Δ*G*^o^_ads_ (kJ mol^−1^)
Weight loss	0.99729	3.49 × 10^5^	−41.57
Tafel	0.99982	1.99 × 10^6^	−45.89
EIS	0.99973	1.07 × 10^6^	−44.35

**Table 5:** Quantum chemical parameters of various structures of MID in water, calculated on B3LYP/6-31G(d) level of theory.

Structure	HOMO (eV)	LUMO (eV)	Δ*E* (eV)	*µ* (Debye)	*χ* (eV)	*η* (eV)	*σ* (eV^−1^)	Δ*N*
closed-ring	−6.039	−1.693	4.346	4.653	3.866	2.173	0.460	0.141
open-ring	−5.799	−2.213	3.587	8.600	4.007	1.793	0.558	0.132
protonated	−6.582	−2.347	4.236	16.414	4.465	2.118	0.472	0.004

**Table 6:** Comparative data on the corrosion parameters, inhibition efficiency, free energy of adsorption, and energy gap of different molecules used as inhibitors for copper corrosion.

Corrosive Media	Inhibitor/Concentration	*i*_corr_ (µA cm^−2^)	*R*_P_ (Ω cm^2^)	*E* (%)	Δ*G*^o^_ads_ (kJ mol^−1^)	Δ*E* (eV)	Ref.
HNO_3_ 0.1 M	Midazolam drug/(imidazo-benzodiazepine)	8.7	2540	92.9	−45.89	4.207	This work
HNO_3_ 0.5 M	Levetiracetam drug/300 ppm (pyrrole)	154.7	167.3	91.7	−19.24	4.1435	[34]
HCl 1.0 M	Metronidazole drug/1 mM (imidazole)	10.3	3012.6	91.8	n/a	4.583	[46]
Acid rain sol.	Ibuprofen drug/10 mM	0.287	29,300	97.2	−31	9.31	[47]
HNO_3_ 0.1 M	1-Methylimidazole/1 mM	2.0	8772	76.0	−34.818	6.4889	[48]
HNO_3_ 1.0 M	L-methionine sulfone/5.0 mM	1.11	10,384	90.7	n/a	3.776	[10]
HNO_3_ 1.0 M	Triazine derivative/0.1 mM	13.95	415.6	91.0	−44.7	1.39	[49]
HNO_3_ 2.0 M	3-amino-1,2,4-triazole/10 mM	95.3	2269	73.9	−29.95	5.7528	[50]
HNO_3_ 2.0 M	Quinoxaline derivative/1 mM	62.2	n/a	82.9	n/a	3.259	[5]
HNO_3_ 2.0 M	2-(2-benzimidazolyl)-4(phenylazo) phenol/1 µM	235	45.08	96.8	−48.75	3.189	[41]
HNO_3_ 2.0 M	Spiropyrazole derivative/100 mg/L	17.12	14.98	89.9	−11.28	7.837	[51]
NaCl 3.5%	5-phenyl-1,3,4-thiadiazole-2-thiol/100 mg/L	0.14	122,000	97.5	−37.5	4.835	[33]
NaCl 3.5%	Benzodiazepine derivative/1 mM	110	8183	96.0	−47	n/a	[3]

## Data Availability

All data contained within the article.
